# Immune enhancement activities of silk lutein extract from *Bombyx mori* cocoons

**DOI:** 10.1186/0717-6287-47-15

**Published:** 2014-04-28

**Authors:** Porkaew Promphet, Sirirat Bunarsa, Manote Sutheerawattananonda, Duangkamol Kunthalert

**Affiliations:** Department of Biology, Faculty of Science, Naresuan University, Phitsanulok, 65000 Thailand; Department of Microbiology and Parasitology, Faculty of Medical Science, Naresuan University, Phitsanulok, 65000 Thailand; School of Food Technology, Institute of Agricultural Technology, Suranaree University of Technology, Nakhon Ratchasima, 30000 Thailand; Centre of Excellence in Medical Biotechnology, Faculty of Medical Science, Naresuan University, Phitsanulok, 65000 Thailand

**Keywords:** Antibody production, BALB/c mice, Immune function, Interleukin-2, Natural killer cells, Silk lutein extract

## Abstract

**Background:**

Declining immune function poses an important clinical challenge worldwide and supplementation with natural products that possessing immune enhancing properties is a promising approach for preventing or delaying immune function decline. Cocoons from yellow silkworms are a significant source of lutein, and this unexplored silk extract could be a viable alternative source for dietary lutein. This study assessed immunomodulatory activities of the silk lutein extract. Female BALB/c mice orally received lutein, either as silk or marigold extracts (10 or 20 mg/kg daily), or vehicle only (1% tween 80 in PBS pH 7.4) for 4 weeks. Natural killer (NK) cell activity, specific antibody production, lymphocyte subpopulations, mitogen-induced lymphocyte proliferation, and cytokine production were examined.

**Results:**

Silk lutein extract increased NK cell activity, and the effect was dose-related whereas marigold lutein extract was ineffective. Silk lutein extract dose-dependently enhanced antibody production in pre-immunized mice but marigold lutein extract had no effect. Feeding with silk lutein extract increased the populations of CD3+ and CD4 + CD3 + cells. Silk lutein extract also stimulated concanavalin A- and lipopolysaccharide-induced proliferations of T and B lymphocytes, respectively. Moreover, silk lutein extract increased IL-2 and IFN-γ production while the effect of marigold lutein extract was undetectable.

**Conclusions:**

Together, silk lutein extract enhanced both innate and adaptive immune functions. This preparation may prove to be an effective supplement for strengthened immunity.

## Background

Immune functions are indispensable as they are the host defenses against infections and cancers, and therefore play a crucial role in maintaining health. Declining immune functions that occur as a result of aging, chronic illnesses, physical and mental stress or unhealthy lifestyles is a major clinical problem globally. Supplementing the immune system with natural products that possess immune enhancing activities is a promising approach for preventing decline of immune functions. Nonprovitamin A carotenoids, notably lutein, have attracted great interest over the decades [1]. The xanthophyll lutein has been noted for its importance in preventing age-related macular degeneration [2–4], cataracts [5], UV-induced skin damage [6], and tumor growth [7–9]. Accumulated evidence to date has also interestingly indicated that lutein has a role in regulating immune functions [1]. For instance, it increases phytohemagglutinin (PHA)-stimulated lymphocyte proliferation [8] and enhances antibody production in response to T-dependent antigen in murine spleen cells [10]. Studies that used animals, which are inefficient converters of carotenoids to vitamin A, also supported the immune regulating role of lutein [11, 12]. Dietary lutein supplementation has been shown to stimulate both humoral and cellular immunity in cats, dogs as well as zebra finches [11–13]. The use of lutein has been increasingly recommended by some physicians as well as more generally used by the public as a dietary supplement [14–16].

Human cannot synthesize lutein and uptake is therefore dependent on the consumption of diet including certain fruits, leafy green vegetables and egg yolks [17]. Lutein used in most studies as well as that contained in most commercial dietary supplements is obtained from marigold flowers. Alternative sources of lutein have, however, continuously been investigated [18, 19]. Yellow cocoons of silkworms, *Bombyx mori* have gathered increasing attention on the grounds of studies that reported to contain a significant amount of the carotenoid pigments [20]. Up to 88% of carotenoids in yellow silk cocoons is the xanthophyll lutein [21]. Providing that, the silk lutein extract from yellow cocoons could alternatively become a valuable dietary resource and may expand uses of lutein in the field of medicine, especially in immune modulating therapies.

The present study was therefore designed to investigate the effects of such yellow silk lutein extract in modulating immune functions that require both innate and adaptive systems to work in concert. The effects of an equivalent content of lutein derived from marigold extract were also examined. While previous research showed actions only on the adaptive arm, this study also examined innate immune responses to silk lutein extract, and it was found that there was selectively increased NK cell and T and B lymphocyte activities.

## Results

### Body weights, and spleen and thymus indices

Daily oral administration of lutein extracts either from silk cocoons or from marigolds produced no signs of ill-health (behavior, body coat, feces, etc.), no mortalities, and no differences in body weights. In addition, there were no differences in the spleen and thymus indices between the control and treatment groups throughout the study (data not shown).

### Effect on natural killer cell activity

**Figure 1:**
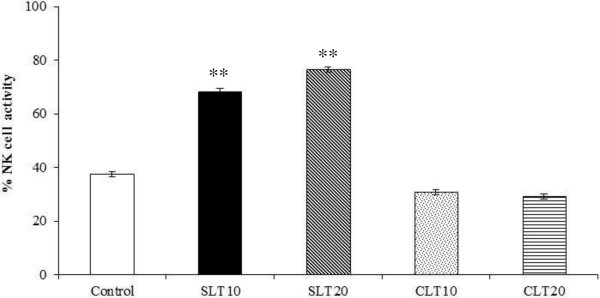
**Effect of silk lutein extract and marigold lutein extract on NK cell activity.** Splenic cells were isolated from BALB/c mice fed lutein extracts from silk cocoons or marigolds daily for 2 weeks and cultured with YAC-1 cell line at a ratio of 100: 1. After 20 hours in a 37°C and 5% CO_2_ incubator, activity of NK cells was determined by MTT assay. Values represent means ± SEM. ** P < 0.01 compared to the control. SLT10, SLT20; silk lutein extract 10 and 20 mg/kg groups, CLT10, CLT20; marigold lutein extract 10 and 20 mg/kg groups.

The oral administration of silk lutein extract (10 and 20 mg/kg) for 2 weeks clearly increased (P < 0.01) the activity of NK cells, and these effects appeared to be dose related as demonstrated in Figure 1. In contrast, none of the samples from the animals treated with marigold lutein extract appeared to show any change.

### Effect on splenic lymphocyte subpopulations

**Figure 2:**
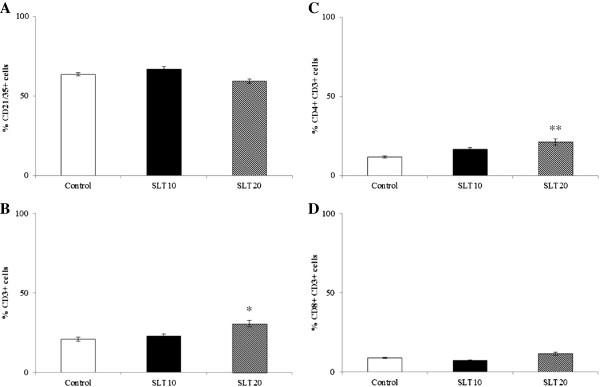
**Population changes in lymphocyte subsets in BALB/c mice fed silk lutein extract daily for 4 weeks.** Splenic single cells were isolated and labeled with fluorescently conjugated monoclonal antibodies specific for the mouse B cell markers CD21/35 **(A)**, and the mouse T cell markers CD3 **(B)**, CD4 **(C)** and CD8 **(D)**. The percentages of lymphocyte subsets were quantitated by Flow cytometry. Values represent means ± SEM. * P < 0.05; ** P < 0.01 compared to the control. SLT10, SLT20; silk lutein extract 10 and 20 mg/kg groups.

Alterations in the percentages of lymphocyte subsets were observed after lutein extract administration and illustrated in Figure 2. Oral administration of silk lutein extract consecutively for 4 weeks did not influence the percentages of CD21/35+ B cells and CD8 + CD3 + T cytotoxic (Tc) (Figure 2A and 2D). However, significant increases in the percentage of CD3+ total T cells (P < 0.05) and CD4 + CD3 + T helper (Th) cells (P < 0.01) were detected in mice fed 20 mg/kg silk lutein extract compared to the control (Figure 2B and 2C). In contrast, mice in the marigold lutein extract treated groups did not show any differences in total T and Th populations throughout the 4-week period and this have previously been described elsewhere [22].

### Effect on mitogen-induced lymphocyte proliferation

**Figure 3:**
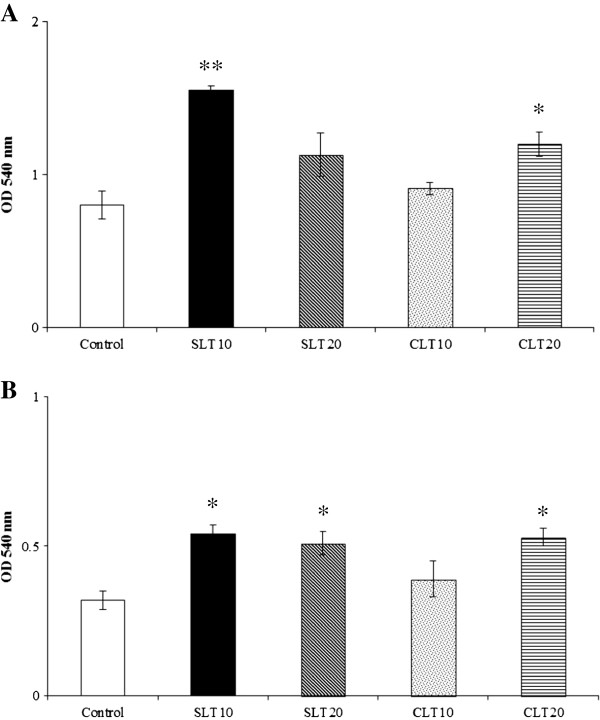
**Effects of silk lutein extract and marigold lutein extract on** ***in vitro*** **lymphocyte proliferation.** Splenic cells were isolated from BALB/c mice fed lutein extracts from silk cocoons or marigolds daily for 4 weeks and cultured in the presence of ConA **(A)** or LPS **(B)** at 37°C in a 5% CO_2_ incubator. After 20 hours, MTT assay was performed. Values represent means ± SEM. * P < 0.05; ** P < 0.01 compared to the control. SLT10, SLT20; silk lutein extract 10 and 20 mg/kg groups, CLT10, CLT20; marigold lutein extract 10 and 20 mg/kg groups.

Silk lutein extract (10 and 20 mg/kg) significantly enhanced the ConA-induced proliferative response of splenic lymphocytes, but this did not appear to be dose related (Figure 3A). Marigold lutein extract (20 mg/kg) also showed such proliferative effects. For LPS (2.5 μg/ml) as the challenge, increased (P < 0.05) lymphocyte proliferative responses were observed in mice fed silk lutein extract (10 and 20 mg/kg) and marigold lutein extract (20 mg/kg) (Figure 3B). Furthermore, the magnitudes of the lymphocyte proliferations were found to be similar in all of these lutein extract treated groups.

### Effect on cytokine production

The production of cytokines measured from the supernatants of ConA-stimulated splenic cultures from mice fed lutein extracts from silk cocoons or marigolds are illustrated in Table 1. Mice fed 10 and 20 mg/kg silk lutein extract had significantly increased (P < 0.05 and P < 0.01, respectively) levels of IL-2 compared to the control mice. Elevated levels of IFN-γ were also observed in the silk lutein extract treated groups compared to the controls. In contrast, the marigold lutein extract treated animals (10 and 20 mg/kg) showed no differences in such cytokine production. No treatment differences in the levels of IL-4 and IL-10 cytokines were found after the 4-week period (data not shown).

**Table 1:** ***In vitro*** **cytokine production by splenic cells from BALB/c mice fed with lutein extracts from silk cocoons or marigolds daily for 4 weeks** ^**1**^

Treatment group	Cytokine concentration (pg/ml)	
	IL-2	IFN-γ
Control	102.4 ± 25.4	1874.4 ± 304.0
SLT10	187.1 ± 18.9*	2568 .2 ± 233.5
SLT20	219.3 ± 11.9**	2265.6 ± 162.3
CLT10	97.5 ± 15.2	1037.8 ± 274.1
CLT20	97.5 ± 3.7	1787.8 ± 188.9

^1^ Splenic cells from mice fed with lutein extracts from silk cocoons or marigolds were cultured with ConA at 37°C in 5% CO_2_ atmosphere. After 72 hours, supernatants were harvested and analyzed by sandwich ELISA. Values represent means ± SEM. * P < 0.05; ** P < 0.01 compared to the control. SLT10, SLT20; silk lutein extract 10 and 20 mg/kg groups, CLT10, CLT20; marigold lutein extract 10 and 20 mg/kg groups.

### Effect on specific antibody production

**Figure 4:**
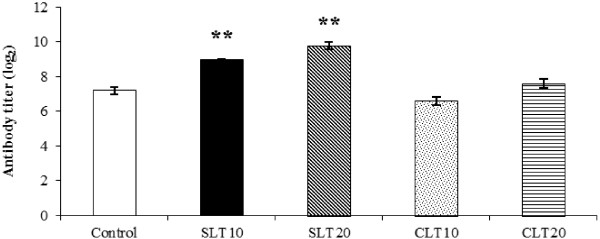
**Levels of anti-sheep erythrocyte antibodies in BALB/c mice fed silk lutein extract or marigold lutein extract.** A day after cessation of treatment, mice were intraperitoneally injected with sheep erythrocytes as antigens. Second immunization was carried out on day 14 and blood samples were collected 7 days after the boost. Specific anti-sheep erythrocytes antibodies were determined by hemagglutination. Values represent means ± SEM. ** P < 0.01 compared to the control. SLT10, SLT20; silk lutein extract 10 and 20 mg/kg groups, CLT 10, CLT 20; marigold lutein extract 10 and 20 mg/kg groups.

The effect of lutein extracts from silk cocoons or marigolds on the production of specific antibodies was determined by measuring anti-sheep erythrocyte antibody titers. As shown in Figure 4, mice fed 10 or 20 mg/kg silk lutein extract had significantly higher (P < 0.01) concentrations of anti-sheep erythrocyte antibodies than the control mice. Administration of marigold lutein extract at the dosages of 10 or 20 mg/kg did not result in elevated antibody titers.

## Discussion

By assessing the key immune parameters in a BALB/c mouse, an appropriate animal model for immunomodulatory study of carotenoids [23], the present work demonstrated that silk lutein extract significantly enhanced the immune functions, both innate and adaptive arms. The antibody response to a specific antigen (the sheep erythrocytes herein) was clearly increased by the silk lutein extract while the marigold lutein extract had no effect. For the specific immune cell responses, a more complex picture emerges with the yellow silk lutein extract. (i) For NK cells, the enhancements of NK activity were apparent as early as 2 weeks after the initiation of lutein extract administration. To our knowledge, this is the first time such lutein-containing extract stimulation of NK cells has been identified. Since NK cells are anti-tumor and antiviral hunter-killers, our findings would suggest the potential for silk lutein extract to augment NK cell surveillance. (ii) There was an indication of an elevated percentage of total T cells, which was reflected in the Th subset but not the Tc; and (iii) T cells secreted IL-2 and IFN-γ and their increased levels were consistent with the lymphocyte stimulation.

Mitogen-induced lymphocyte proliferation is a powerful indicator of adaptive immune functions. In the present study, silk lutein extract strengthened the splenic lymphoproliferative response to ConA after 4 weeks of administration. Such lymphocyte stimulation accords with the increased CD3+ total T and CD4 + CD3+ Th cell counts (Figure 2). Interestingly, only silk lutein extract stimulated the production of IL-2. Therefore, enhanced lymphocyte proliferation by silk lutein extract is likely attributable to the increases in total T and Th subpopulations as well as to the production of IL-2. In contrast, the marigold lutein extract in our study did not alter the IL-2 level despite stimulated ConA-induced lymphocyte proliferation. Similarly, increased lymphocyte proliferation without IL-2 production has been reported in cats, dogs and mice given marigold lutein extract [8, 11, 12]. In this regard, increased lymphoproliferative response may be mediated by the alterations in the expression of surface molecules such as major histocompatibility complex class II and IL-2 receptors, which are responsible for antigen presentation and clonal expansion, respectively. Taken together, the results of this study suggest that silk lutein extract can enhance T cell functions, perhaps differently from the marigold lutein extract.

Upon stimulation with sheep erythrocyte antigen, the levels of anti-erythrocyte antibodies in plasma were elevated in mice fed silk lutein extract. Although B cell numbers appeared unchanging, it is more likely that the plasma cell functions may have been promoted. The increased concentration of antigen-specific antibodies would thus indicate the enhancement activity of the silk lutein extract on humoral immune system responding to pathogens and other foreign intrusions. This finding is also of clinical significance because silk lutein extract may serve as an adjuvant to help stimulate the antibody production for routine vaccinations. Discovery of new non-toxic adjuvants within the collections of natural products has recently been put forward as an urgent need for producing more efficacious vaccines [24]. While marigold lutein extract failed to show increased antibody titers for mice in this study, such effects have been observed in dogs [11] and cats [12]. Lutein given to dogs and cats was pre-mixed with soybean oil [11] or basal diet containing high fat [12] whereas the mice in this study were fed only with a suspension of lutein. Besides, lutein dosage, species of animals, challenged antigens, and immunization schedules could also possibly contribute to such differences.

The contents of lutein either from silk or marigold extracts given to the animals herein were equivalent. Nevertheless, there are certain differences in the silk and marigold lutein extract preparation in this study. Lutein in the silk extract was predominantly in a free unesterified form whereas most commercial marigold lutein is present in a diesterified form [25]. The silk, but not marigold, extract also contained fats including free fatty acids plus other insoluble wax materials. Until now, it is accepted that in order to achieve its potential beneficial health effects, the carotenoid lutein must be efficiently absorbed and carried to its target tissues. Among dietary factors that influence such bioavailability, fat seems to exert the greatest impact on lutein absorption. This is supported by the fact that the absorption efficiency of lutein increases when the amount of fat in the diet increases [26–30]. Available data have also demonstrated that the bioavailability of lutein is much more sensitive to a fat containing diet than other carotenoids such as β-carotene. It was shown that 3 g fat were sufficient for optimum absorption of α- and β-carotene whereas higher amounts of fat (36 g) were required for optimum absorption of lutein [26]. Recent studies where types of fatty acids on lutein bioavailability was assessed have also revealed that dietary fats rich in saturated fatty acids lead to a higher bioavailability of lutein [31]. Regarding to this, the presence of fats in the silk extract may promote solubilization and perhaps aid stability of lutein, resulting in the efficient absorption of lutein. Since palmitic and steric acids were the predominant saturated fatty acids identified in the silk extract, these fatty acids would also enhance such a critical step for great bioavailability of lutein. Moreover, the oleic acid in the silk extract may augment such action as this fatty acid has also been reported to modulate the absorption of dietary lutein [32]. Since the unesterified form of lutein has greater bioavailability [33–37], this could additionally explain the preferable effects of the silk lutein extract, in comparison to marigold lutein extract, in the present study. The fact that lutein from the silkworm *B. mori* usually presents in a protein-binding form [38, 39] and that bound protein was removed during the extraction process, there is a possibility that the binding site of such lutein remains active and preferentially acts on the target tissues, in different manners to the esterified marigold lutein. Although mechanisms responsible for this remain to be clarified, the evidence described herein could explain, to a certain degree, the differences of immune enhancing outcomes between silk and marigold lutein extracts. The findings in this study support the requirement of fats for lutein, and highlight the importance of the sufficiently suitable fats in silk extract preparation to facilitate absorption before the lutein could efficiently exert its maximal immune activity.

## Conclusions

In sum of the data, the present study demonstrated that a package of the yellow silk cocoon extract containing naturally occurring lutein and fats was capable of efficiently enhancing both innate and adaptive immune functions. Such silk lutein extract may prove superior to existing plant lutein extract for strengthened immunity and consequent health improvement.

## Methods

### Silk lutein extract preparation

**Figure 5:**
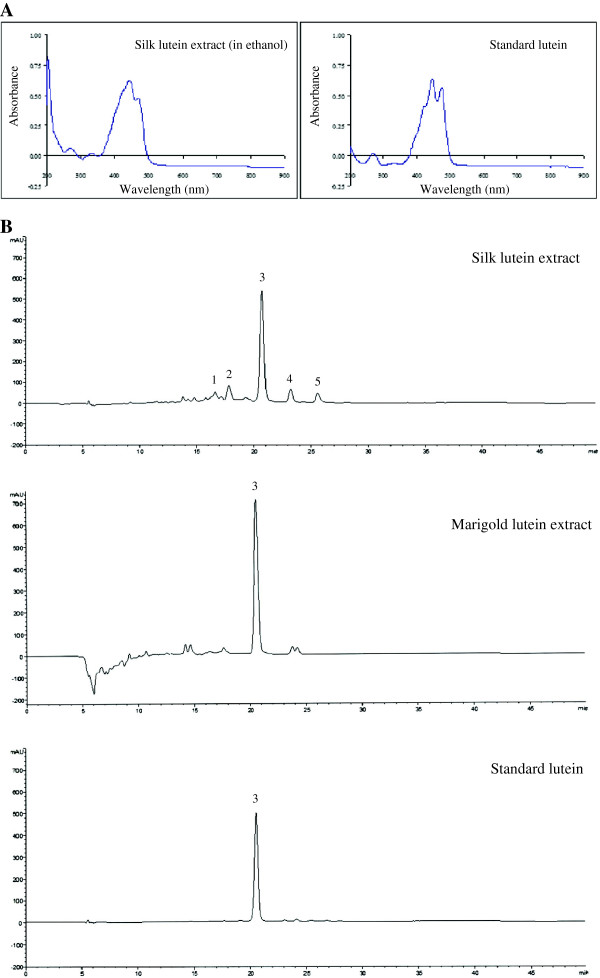
**Fingerprints of silk lutein extract, marigold lutein extract and standard lutein (Sigma). (A)** UV/VIS absorption spectra **(B)** HPLC chromatograms at 445 nm. HPLC was conducted using a LiChrospher®100 reversed-phase C30 column as stationary and acetonitrile/methanol (9:1, v/v) and ethyl acetate as mobile phases. Peaks: 1, (13-Z)-lutein; 2, (13′-Z)-lutein; 3, (all-E)-lutein; 4, (9-Z)-lutein and 5, (9′-Z)-lutein.

Silk lutein extract was prepared according to the method described in the patent with international publication number WO 2012/091683 A1. Yellow cocoons from the Nangnoi strain of Thai silkworms (*Bombyx mori*) were first soaked in distilled water at a ratio of 1:30 and then degummed at 121°C for 15 minutes to partially remove glutinous silk protein sericin. After being heated, degummed cocoons and degumming solution were separated off, leaving degummed cocoons. Next, the degummed cocoons were fourfold extracted with 30 ml of hexane/ethanol/ethyl acetate (3:2:1, v/v/v) with 0.1% butylated hydroxytoluene (w/v) until colorless. The resulting solution was collected and kept in an amber glass sample bottle at 4°C. Then, it was partitioned into non-aqueous phase and an aqueous phase by adding 100 ml of aqueous sodium chloride at 10% (w/v) into the organic mixture solution. The supernatant was separated from the aqueous phase and evaporated to dryness under vacuum at temperature ≤ 35°C. The dried residue was dissolved in hexane/ethyl acetate (3:1, v/v) to obtain a preferred volume of 5 to 10 ml and then filtered through a 0.45 μm PTFE syringe filter to obtain lutein extract. Analysis using UV/VIS spectrophotometer (Libra S22, Biochrom Ltd., UK) revealed the absorption spectrum for lutein in the resulting silk extract, as compared with the authentic lutein standard (Figure 5A). Carotenoids and their compositions in the silk extract were quantified by reversed-phase high-performance liquid chromatography (HPLC; Agilent HP 1100 series). The silk extract contained lutein 5.4% w/w and this represented a major constituent, accounting for 91.26%, of total carotenoids in such extract. The HPLC chromatogram of the silk lutein extract, along with that of the standard lutein, is shown in Figure 5B. As analyzed by gas chromatography, the silk lutein extract also contained free fatty acids 10.41% w/w while the remainder was insoluble unidentified wax materials. The compositions of fatty acids in the silk lutein extract are presented in Table 2. Marigold lutein extract from *Tagetes erecta* flowers was purchased from P.R. China through TTK Science (Bangkok, Thailand). The marigold extract contained lutein 97.2%, as specified by the manufacturer. Analysis by liquid chromatography-mass spectrometry (LC-MS) using Ultraflex III TOF/TOF (Bruker Daltonik, GmbH) further confirmed identity of lutein in the silk and marigold extracts. LC-MS analysis also displayed Maldi-TOF MS spectra corresponding to kaempherol and quercetin in the marigold extract whereas these flavonoids were not detected in the silk lutein extract (data not shown).

**Table 2:** **Fatty acid compositions of silk lutein extract** ^**1**^

Fatty acid	g/g of fat	%	Fatty acid	g/g of fat	%
Capric acid	0.0005	0.05	Alpha Linolenic acid	0.0056	0.56
Lauric acid	0.0010	0.10	Arachidic	0.0027	0.27
Myristic acid	0.0013	0.13	cis-11-Eicosenoic acid	0.0002	0.02
Pentadecanoic acid	0.0002	0.02	c9, t11	0.0002	0.02
Palmitic acid	0.0197	1.97	c9, c11	0.0006	0.06
Palmitoleic acid	0.0009	0.09	cis-11,14-Eicosedienoic acid	0.0001	0.01
Stearic acid	0.0161	1.61	cis-8,11,14-Eicosatrienoic acid	0.0037	0.37
Elaidic	0.0007	0.07	Behenic	0.0018	0.18
Oleic acid	0.0290	2.90	cis-4,7,10,13,1 6,19-Docosahexaenoic acid	0.0017	0.17
Linoleic acid	0.0137	1.37	Erucic	0.0015	0.15
Gamma-Linolenic acid	0.0005	0.05	Lignoceric	0.0024	0.24

^1^ Fatty acid compositions were analyzed by gas chromatography (Hewlett Packard GC system HP6890 A; Hewlett Packard, PA) equipped with a 100 m × 0.25 mm fused silica capillary column (SP2560, Supelco Inc, Bellefonte, PA). Injector and detector temperatures were set at 250°C. The column temperature was kept at 4°C for 4 min, then increased at 13°C/min to 175°C and held at 175°C for 7 min, then increased at 4°C/min to 215°C and held at 215°C for 17 min.

Based on lutein concentration determined by HPLC, the silk lutein extract solubilized in dimethylsulfoxide (DMSO) was dissolved with 1% tween 80 in phosphate buffered saline (PBS) pH 7.4 to achieve the desired concentrations to be given to the animals. This working lutein solution was prepared weekly and kept at −20°C in sealed containers in the dark under nitrogen gas. Preparation of the marigold lutein extract was performed precisely as described for the silk lutein extract.

### Animals and experimental design

Female BALB/c mice (7 weeks old; 20–23 g) were obtained from the National Laboratory Animal Center, Mahidol University, Thailand. Animals were housed in polycarbonate cages maintained at 25 ± 1°C with a 12-h dark: light cycle and had free access to sterile water and standard mouse diet (CP Company, Thailand; Table 3). Animal procedures were reviewed and approved by the Animal Research Ethics Committee of Naresuan University, Thailand (Permit Number: 52040007). All animal experiments were conducted in accordance with the institutional guidelines for the care and use of laboratory animals. The mice were randomly assigned to five groups of five animals each and were orally administered via a feeding tube with either: (1) vehicle (1% Tween 80 in PBS pH 7.4; Controls); (2) 10 mg/kg body weight of silk lutein extract (SLT10); (3) 20 mg/kg silk lutein extract (SLT20); (4) 10 mg/kg commercial lutein marigold extract (CLT10; P.R. China); and (5) 20 mg/kg commercial lutein marigold extract (CLT20). These dosages were administered once daily for a period of 4 weeks. Animal body weights were recorded weekly and routine clinical observations were carried out throughout the experiments.

**Table 3:** **Nutritional composition of mice feed**

Component	Amount	Component	Amount
Moisture	12%	Vitamin A	20,000 i.u./kg
Crude protein	24%	Vitamin D	4,000 i.u./kg
Fat	4.5%	Vitamin E	100 mg/kg
Fiber	5%	Vitamin K	5 mg/kg
Calcium	1%	Vitamin B1	20 mg/kg
Phosphorus	0.9%	Vitamin B2	20 mg/kg
Sodium	0.2%	Vitamin B6	20 mg/kg
Potassium	1.17%	Vitamin B12	0.036 mg/kg
Magnesium	0.23%	Niacin	100 mg/kg
Manganese	171 p.p.m.	Folic acid	6 mg/kg
Copper	22 p.p.m.	Biotin	0.4 mg/kg
Zinc	100 p.p.m.	Pantothenic acid	60 mg/kg
Iron	180 p.p.m.	Choline chloride	1,500 mg/kg
Cobalt	1.82 p.p.m.	Metabolizable energy	3,040 kcal/kg
Potassium iodide	1 p.p.m.		
Selenium	0.1 p.p.m.		

Five mice in each group were humanely killed by intraperitoneal injection with an overdose of pentobarbital sodium (Nembutal^R^, Ceva Sante Animale, France; 40–50 mg/kg body weight) on week 2 (for assessment of natural killer cell activity) and week 4 (for other immune parameters) after the initial lutein extract administration. The spleens and thymus were removed and weighed immediately. Their indices were expressed as 100 × spleen or thymus weight/ body weight.

### Splenic single cell preparation

After being removed, the spleens were aseptically saved in a PCM buffer prepared with sterile PBS pH 7.4 containing 7 × 10^−4^ M CaCl_2_, 5 × 10^−4^ M MgCl_2_, 5% (v/v) fetal bovine serum (Gibco, South America), 100 U/ml penicillin, and 100 μg/ml streptomycin (PAA, Pasching, Austria). Single cells were mechanically separated via a cell strainer (BD Falcon, NJ). Erythrocytes were removed by lysis in 0.17 M NH_4_Cl pH 7.65, and the resulting cell suspensions were washed twice with a PCM buffer by centrifugation. Cell pellets were finally resuspended with RPMI-1640 (PAA) containing 10% (v/v) fetal bovine serum, 0.01 M HEPES pH 7.4, 5 × 10^−5^ M β-mercaptoethanol (BioRad, Hercules, CA), 2 mM L-glutamine, 100 U/ml penicillin and 100 μg/ml streptomycin. The viability of the splenic cells was determined by trypan blue dye exclusion using a hemocytometer.

### Natural killer cell activity

Splenic natural killer (NK) cell activity was evaluated by 3-(4,5-dimethylthiazol-2-yl)-2,5-diphenyltetrazolium bromide (MTT) assay as previously described [40, 41] with few modifications. Briefly, splenic cells (effector cells), prepared as described above, and YAC-1 cells (target cells; American Type Culture Collection, Manassas, VA) were incubated together in 96-well flat-bottom plates (Nunc^R^) (effector: target ratio of 100:1) with a total volume of 200 μl in each well. The plates were incubated at 37°C in a humidified 5% CO_2_ atmosphere. After 20 hours, 100 μl of the culture supernatants were removed and 40 μl of MTT (5 mg/ml; Sigma, St. Louis, MO) was added. After an additional 3 hours of incubation, the plates were subjected to an MTT assay [42]. Control wells contained either effector or target cells alone, and all tests were performed in triplicate. The optical density (OD) at 540 nm was determined by using a microplate spectrophotometer (Labsystem iEM Reader MF). The percentage of NK cell cytotoxic activity was calculated according to the formula: {1- [(OD test – OD effector cell control) / OD target cell control]} × 100.

### Splenic lymphocyte subpopulations

The percentages of lymphocyte subpopulations in the spleen were quantified by Flow cytometry. Splenic single cell suspensions (4 × 10^5^ cells) were stained for 30 minutes on ice with fluorescently conjugated antibodies specific for the mouse T cell markers CD3, CD4 and CD8, and for the mouse B cell markers CD21/35. Fluorescein isothiocyanate conjugated anti-mouse CD3e (clone eBio500A2), phycoerythrin (PE) conjugated anti-mouse CD4 (clone RM4-5), PE conjugated anti-mouse CD8a (clone 53–6.7), and PE conjugated anti-mouse CD21/CD35 (clone eBio8D9) were all purchased from eBioscience (San Diego, CA). Appropriate isotype-matched antibodies were used as controls for background staining. Stained cells were washed twice with cold-PBS containing 2% fetal bovine serum and then fixed with 1% paraformaldehyde. Cells were then analyzed with a Becton Dickinson FACScalibur and CellQuest Pro software (Becton Dickinson). A total of 5,000 events were acquired for each analysis.

### Mitogen-induced lymphocyte proliferation

The proliferation of splenic lymphocytes in response to T and B cell mitogens was determined by MTT assay [42]. A total of 2 × 10^5^ splenic cells were cultured in flat-bottom 96-well plates (Nunc^R^) in the presence or absence of Concanavalin A (ConA, 0.5 μg/ml; Sigma) or lipopolysaccharide (LPS, 2.5 μg/ml; Sigma) and incubated at 37°C in a 5% CO_2_ atmosphere. After 48 hours, 20 μl of MTT (5 mg/ml) was added and the plates were incubated for another 4 hours. Supernatant in each well was discarded, and 100 μl of DMSO was added to dissolve the formazan crystals. The OD at 540 nm of each well was measured on a microplate spectrophotometer (Labsystem iEM Reader MF). All assay tests were done in triplicate.

### Cytokine production

The production of regulatory cytokines caused by splenic lymphocytes was determined by sandwich enzyme-linked immunosorbent assay (ELISA) using Ready-Set-Go for mouse IL-2, IFN-γ, IL-4 and IL-10 (eBioscience). All assay procedures were performed according to the manufacturers’ instructions. A total of 2 × 10^5^ splenic cells were cultured in flat-bottom 96-well plates (Nunc^R^) in the presence of Con A (0.5 μg/ml for interferon-γ (IFN-γ), interleukin-4 (IL-4) and interleulin-10 (IL-10), and 2.5 μg/ml for interleukin-2 (IL-2). After incubating the cells at 37°C in 5% CO_2_ atmosphere for 72 hours, the cultured supernatants were collected and stored at −20°C until assayed. The detection limits were 2, 15, 4, and 30 pg/ml for IL-2, IFN-γ, IL-4, and IL-10, respectively.

### *In vivo* antibody response

Humoral immune response was assessed through an immunization protocol with sheep erythrocytes as antigens. A day after ceasing treatment, the mice were intraperitoneally injected with 2 × 10^8^ erythrocytes in 0.2 ml saline solution. A second immunization was carried out on day 14 and blood samples were collected 7 days after the boost. Specific anti-sheep erythrocytes antibodies were determined by hemagglutination. The reciprocal serum dilution with ≥ 50% agglutination was considered to be the titer.

### Statistical analysis

Data are expressed as mean ± SEM. The differences among groups were evaluated by one-way analysis of variance (ANOVA), followed by Tukey’s Honestly Significant Difference test. All statistical analyses were performed with SPSS version 11.5 (SPSS Inc., Chicago, IL). Differences were considered to be significant at P < 0.05.
